# MicroRNA mediators of early life stress vulnerability to depression and suicidal behavior

**DOI:** 10.1038/s41380-019-0597-8

**Published:** 2019-11-18

**Authors:** Lauren Allen, Yogesh Dwivedi

**Affiliations:** Department of Psychiatry and Behavioral Neurobiology, University of Alabama at Birmingham, Birmingham, AL USA

**Keywords:** Depression, Neuroscience

## Abstract

Childhood environment can have a profound impact on brain structure and function. Epigenetic mechanisms have been shown to play a critical role in adaptive and maladaptive processes by regulating gene expression without changing the genome. Over the past few years, early life stress (ELS) has been established as a major risk factor for major depression and suicidal behavior along with other psychiatric illnesses in adulthood. In recent years, the emergence of small noncoding RNAs as a mega controller of gene expression has gained attention for their role in various disease processes. Among various noncoding RNAs, microRNAs (miRNAs) are the most studied and well characterized and have emerged as a major regulator of neural plasticity and higher brain functioning. More recently, although limited in number, studies are focusing on how miRNAs can play a role in the maladaptive processes associated with ELS both at adolescent and adult age and whether these processes are critical in developing depression and suicidal behavior. In this review, we critically evaluate how postnatal ELS relates to abnormalities in miRNA expression and functions from both animal and human literature and draw connections from these findings to depression and suicidal behavior later in life.

## Introduction

Early life is a sensitive time period for brain development where adverse experiences can have far-reaching consequences. Nearly one million cases of child maltreatment are reported in the United States each year, which includes reports of neglect, physical abuse, and sexual abuse [1]. Although not all stress or even all early life stress (ELS) is maladaptive, ELS has been reliably associated with poor life outcomes including heart disease [2], cancer [3], and various psychopathologies including major depression and suicidal behavior [4]. Other types of early life trauma, such as parental loss and natural disaster have also been linked to increased rates of adult-onset depression [5, 6]. Although major depressive disorder (MDD) is not homogenous, most often it is associated with stress as a predisposing or precipitating event. Globally, the lifetime prevalence of MDD varies by country with an overall rate of 3% [7] and 15% in the U.S. adult population [8], costing approximately $100 billion annually [9]. Though antidepressant (AD) use has increased over time [10], both response to and remission rates after using ADs remain low [11–13]. Although our understanding of the neurobiological underpinnings of depression and other psychiatric disorders has substantially improved over the last 2–3 decades, the etiopathology of MDD, and subsequently the optimal therapeutic solution, remains obscure. Moreover, ELS and MDD are both linked to an increased risk of suicide [14, 15]. It is evident that early life adversity poses a significant public health risk.

Recently, the contribution of early life experiences to stress and depression vulnerability has received tremendous attention [16]. In a broad context, stress elicits a host of biological responses including neurochemical cascades in the hypothalamic–pituitary–adrenal (HPA) axis [17] and immune reactions [18], which can subsequently alter neuronal connectivity and signaling [19] as well as brain matter density [20]. In resilient adults, chronic stress promotes adaptation over time through dendritic remodeling in the hippocampus, amygdala, and prefrontal cortex (PFC) [21], yet in the case of susceptible individuals, these adaptive changes may not be reversible after stress is removed [22]. In order to enact these changes, the environment interacts with gene function without directly affecting the genome itself through epigenetic modifications like methylation, histone modifications, and noncoding RNAs [23]. These changes have all been identified in patients with major depression and those who have committed suicide (see [24, 25] for review) with some focus on ELS [23]. The adolescent brain is significantly more structurally plastic and readily encodes environmental experience into structural and functional changes through epigenetic programming [26]. Because development is cumulative, ELS has the potential to cause widespread alterations in brain function that persist over the lifetime [27]. It is still not clear which factors associated with ELS are the most detrimental for later life outcomes or exactly how these events can have such long-lasting effects on brain functions. Furthermore, it has been proposed that depression, subsequent to ELS, comprises a subset of patients with unique etiopathology who would benefit from different treatment or preventative medicine as compared with other patients [28, 29]. Further understanding of the molecular neurobiology associated with ELS will bring us closer to identifying vulnerable populations and developing effective treatments.

Adverse prenatal and postnatal experiences have both been associated with later onset depression [30–32]. However, the neurobiological and epigenetic mechanisms by which these stressors lead to adult stress-susceptibility seems uniquely linked to their developmental timing. Physical and psychological stressors experienced by the mother can indirectly affect the fetus prenatally as a direct neurochemical stressor via the placenta. Provencal and Binder [33] reported that alterations in gene function and epigenetic changes in the placenta which lead to increased placental permeability to glucocorticoids (GCs) are specific outcomes of prenatal stress. While both pre- and postnatal stress have been associated with epigenetic modifications via upstream signaling mechanisms including GC receptor (GR) activity, with prenatal stress these effects are restricted to the uterine environment during gestation [33]. In contrast, postnatal ELS constitutes a variety of stressor types (i.e. physical or psychological) that are directly experienced until adulthood.

The current review aims to identify noncoding RNAs, specifically microRNAs (miRNAs) as key to stress susceptibility as a result of these directly experienced postnatal stressors. In addition, this review will critically examine the role of ELS-associated miRNAs across the existing depression and suicide literature.

## Overview of miRNAs

**Fig. 1:**
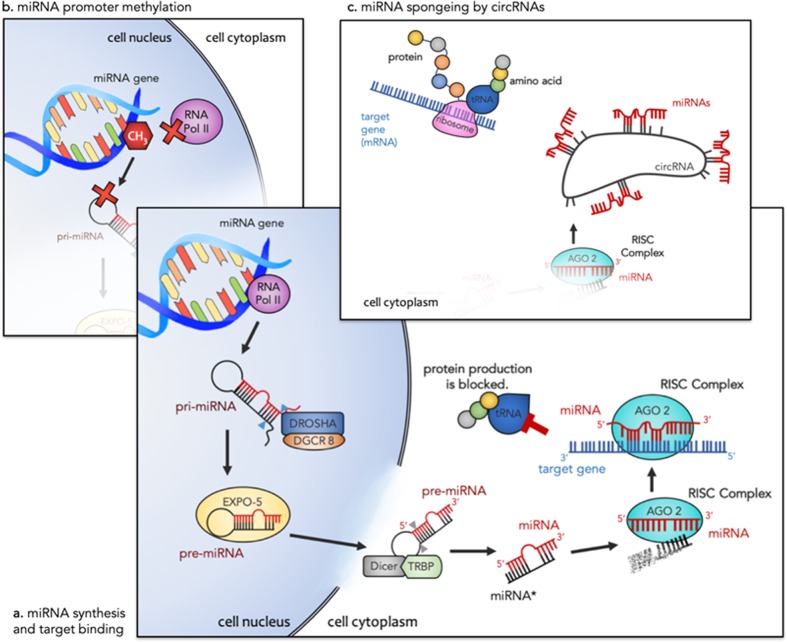
**a** First RNA polymerase II transcribes the miRNA gene resulting in a pri-miRNA with a hairpin loop structure. This structure is cleaved by DROSHA and DGCR8 (blue arrows) into a pre-miRNA and transported out of the cell by EXPO-5. Dicer and TRBP cleave away the loop structure (gray arrows) leaving a miRNA-miRNA* duplex. AGO 2 loads the mature miRNA (red), forming the RISC complex, and the miRNA* strand (black) is degraded. RISC can bind to specific gene targets and lead to translational repression. **b** Methylation at the miRNA gene promoter region can reduce transcription of pri-miRNAs by RNA Pol II. This results in decreased production of mature miRNAs and altered downstream repression of their target genes. **c** In the presence of compatible circRNAs, there is competition for miRNA binding. Each circRNA can have multiple binding sites for a single miRNA effectively reducing miRNA-target gene interactions and their associated translational repression. As a result, both methylation and circRNAs can promote protein production. Abbreviations: miRNA (microRNA), RNA Pol II (RNA Polymerase II), pri-miRNA (primary miRNA), pre-miRNA (precursor miRNA), EXPO-5 (exportin-5), TRBP (Tar RNA-binding protein), RISC (RNA-induced silencing complex), AGO 2 (argonaute), tRNA (transfer RNA), CH_3_ (methyl group), mRNA (messenger RNA), circRNA (circular RNA)

The environment alters a host of cellular functions throughout the body via a set of well programmed molecular mechanisms known collectively as epigenetics. Being an integral part of the epigenetic machinery, various modifiers (including noncoding RNAs, methylated cytosine, and chromatin remodelers) have gained recognition in the past decades for their role in development, synaptic plasticity, and cell death and proliferation [34, 35]. MiRNAs, a class of small noncoding RNAs, have recently been implicated in ELS [36, 37] and several mental illnesses [38–41]. Their role in neuronal development and brain physiology [35] has made them a strong candidate for the study of psychiatric disorders affected by ELS. MiRNAs are short sequences of nucleotides (~22 nucleotides long) that have the direct ability to post-transcriptionally modify the cellular stability of messenger RNA (mRNA) thereby altering subsequent protein production. Regardless of their association with any disease or disorder, individual miRNAs can have hundreds of mRNA targets with varying functions making them global modulators of gene expression [34]. Within cell nuclei, right after transcription, primary miRNAs (pri-miRNAs) are cleaved by Drosha ribonuclease III (DROSHA) and microprocessor complex subunit, DGCR8, into precursor miRNAs (pre-miRNAs), Fig. 1a [34]. Exportin 5 translocates pre-miRNAs to the cytoplasm where they are converted into mature miRNAs by the endoribonuclease, Dicer, and TAR RNA-binding protein (TRBP). The double-stranded miRNA: miRNA* complex is bound by an Argonaute protein. Argonaute selects one strand as the mature miRNA and the miRNA* strand is degraded. The mature miRNA within the Argonaute protein is known as the RNA-induced silencing complex (RISC) and can readily pair with specific mRNAs [42]. The nucleotide sequence of mature miRNA is complementary to one—or sometimes many—mRNAs and typically bind to the 3′ untranslated region (UTR) of the target mRNA. MiRNA most often block the translation of mRNA into proteins by a process of repression involving the deadenylation of target mRNA. Alternatively, when a miRNA sequence is highly complementary to its target mRNA, it can slice the mRNA causing degradation [34].

### Epigenetic modification of miRNAs

More recently, epigenetic modifications of miRNAs have been investigated, especially via miRNA promoter region methylation [43–46]. This adds another layer of complexity to understanding epigenetic mechanisms of disease etiopathology. Canonical DNA methyltransferases add a methyl group to cytosines in the promoter region of miRNA coding genes in the same way that conventional genes are methylated (Fig. 1b) [45]. Functionally, methylation-induced conformational changes in chromatin structure make DNA inaccessible for gene transcription—in this case the gene codes for a pri-miRNA [47]. In this way, methylation at the promoter region of a miRNA can repress miRNA expression. Another epigenetic modifier, long noncoding RNA (lncRNA), has been gaining attention in the study of psychiatric disorders [38]. Preclinical studies have identified transcriptome-wide changes in lncRNAs after repeated social defeat stress [48] and learned helpless depression models [49, 50]. A subset of lncRNAs, circular RNAs (circRNAs), have been described as the “RNA sponge” and serve as master regulators of miRNA expression by binding and inhibiting their function, Fig. 1c [51]. Similarly to miRNA promoter region methylation, circRNAs are inversely related to miRNA expression, subsequently increasing miRNA-target gene expression. Whereas methylation can alter miRNA expression by inhibiting its synthesis, circRNAs regulate miRNA bioavailability by binding to mature miRNAs in the cytoplasm.

## miRNAs and ELS

### Preclinical studies

Animal models have been used to assess causal effects of ELS on the epigenome. By far, the most popular animal model of ELS is maternal separation. In this procedure, neonatal pups are separated from dams for some time each day for the first 2–3 weeks of life. Many studies report behavioral changes after maternal separation [36, 52–54], although there are some mixed findings across specific behavioral tests, sex, and rodent strain [55]. To date, only a handful of studies have explored miRNA changes after maternal separation. Zhang et al. reported that 6 h of daily maternal separation from postnatal days (PND) 1–14 resulted in increased expression of miR-504 in the nucleus accumbens [53]. In animals that also experienced chronic unpredictable stress in adulthood, miR-504 expression was further increased. MiR-504 directly targets the 3′UTR of the dopamine D1 receptor gene (*DRD1*) [56] and *DRD1*-containing neurons have been shown to play an important role in the development of anhedonic behavior in rodents [57]. Furthermore, knockdown of DRD1 in mouse medial prefrontal cortex (PFC) causes an increase in avoidant behavior after social defeat stress [58]. Later, Zhang et al. also reported decreased nucleus accumbens expression of miR-9 in animals who received a combination of maternal separation and chronic unpredictable stress [59]. Likewise, an interaction between both stressors caused the greatest changes in miR-326 expression in both nucleus accumbens and striatum (increased and decreased expression, respectively) [59]. Notably, the 3′UTR of the dopamine D2 receptor (*DRD2*) is a predicted target of miR-326, yet Zhang et al. found a positive relationship between miR-326 and *DRD2* expression [59]. Clearly, further studies are needed to elucidate the mechanism by which miR-326 alters *DRD2* after ELS. Nonetheless, resilience after adult social defeat stress in rats has been inversely correlated with miR-326 expression in the amygdala [60]. Bai et al. [36] reported that the same 6 h maternal separation paradigm increased miR-16 expression in the hippocampus as compared with controls and animals who received chronic unpredictable stress. Although only a few changes in miRNA expression were reported after maternal separation, these studies support the notion that ELS induces susceptibility to later life stress at the epigenome level. Uchida et al. [52] maternally separated rodents for 180 min per day (half of the separation time in Zhang et al. [53, 59]) and found significant increases in depression-like behaviors such as anhedonia in the sucrose preference test and immobility in the forced swim test as well as increases in miR-132, miR-124, miR-9, and miR-29a expression. MiR-124 and −132 are mostly restricted to the nervous system and are key to brain development through their roles in neuronal differentiation (miR-124) [61] and morphogenesis (miR-132) [62]. MiR-9 regulates microglia function through its target HECT domain E3 ubiquitin protein ligase 1 (*HECTD1)* [63] and miR-29a has been implicated in apoptotic pathways following endoplasmic reticulum stress via its target, an apoptosis regulator: myeloid leukemia cell differentiation protein (*Mcl-1)* [64]. In addition, RE1 silencing transcription factor (REST), a transcription factor involved in neuronal differentiation, was upregulated after maternal separation [52]. Overexpression of REST 4 in mice caused increased expression of miR-132, miR-121, and miR-9-3 [52]. REST can also repress expression by binding to RE1 sites, which can be found on the regulatory elements of the corticotropin-releasing hormone gene, *CRH* [65] and brain-derived neurotrophic factor (*BDNF)* [66] genes, among others; both genes are important in stress and depression [67]. The promoter region of miRNAs miR-132, miR-124, miR-9, and miR-29a are each relatively close to an RE1 binding site [65]. Bahi [54] employed an alternative paradigm, where half of the pups in each litter were maternally separated in isolation, while the rest of the pups remained with the dam. In the pups who had been separated, a stereotaxic injection of miR-124 lentivirus into the dentate gyrus increased anxiety-like behaviors in the elevated plus maze and reduced social interaction behaviors along with decreased BDNF mRNA expression. Interestingly, although *BDNF* is a known target of miR-124a and both controls and maternally separated animals received a miR-124 injection, only animals who experienced isolated maternal separation exhibited these changes in *BDNF*. It is possible that ELS causes methylation changes, which may make miRNA targets more available for posttranscriptional modification. Otherwise, these miRNAs, though prevalent throughout the brain, cannot affect mRNA translation.

In contrast to ELS, maternal separation in short bouts of 15 min has also been used to induce increased maternal care behaviors [68, 69]. A recent study found that this augmented maternal care (AMC) decreased expression of DGCR8, part of the miRNA processing machinery, in the hypothalamus and increased levels of miR-488, −144, and −542-5p [69]. AMC also decreased expression of miR-421 and miR-376b-5p. These differentially expressed miRNAs are predicted to target a number of genes relevant to stress signaling pathways and neuronal regulation [69]. In particular, miR-144 is predicted to target Galanin [69], a protein important to the noradrenergic system and responsive to restraint stress in rodents [70]. In panic and anxiety disorder, miR-488 has been shown to regulate proopiomelanocortin, a precursor to the HPA hormone, adrenocorticotropin [71]. Another predicted target of miR-488, arginine vasopressin receptor 1a was recently shown to be downregulated in mice after ELS [72]. In a study by Uchida et al. [52], a similar 15-min maternal separation paradigm was used as a handled control and compared with 180-min maternal separation. Compared with animal-facility-reared controls, 15-min maternal separation did not significantly alter behavior or miRNA expression. No other studies have explored the effects of AMC on miRNA expression.

Other animal models of postnatal ELS are mostly applied in the peri-pubertal time period, near PND-28, in order to approximate teen or adolescent stress. Two independent studies have investigated the effects of chronic variable stress (CVS) on miRNA expression in rodent PFC and basolateral amygdala, respectively [73, 74]. Xu et al. [73] found that CVS increased miR-18a and −124a expression in the basolateral amygdala by PND-55, but at PND-90, miR-18a expression was the same in CVS and control animals. MiR-124a, however, remained significantly increased in adult rats at PND-90. In order to exogenously activate the HPA response via GR, a separate sample of animals without CVS were dexamethasone-treated and exhibited the same patterns of miRNA expression as CVS animals. Using the same CVS paradigm, Xu et al. [75] found similarly increased levels of miR-124a and miR-18a expression in both PFC and hippocampus. This further corroborates the hypothesis that ELS acts to sensitize the HPA axis to later life stress. In addition, administration of RU486, a non-selective GR antagonist, negated the effects of both ELS and dexamethasone administration across miRNA expression, gene expression, and behavioral measures [73] thereby showing that these effects are dependent on GR signaling. Specifically, RU486 returned rearing, crossing, and grooming behaviors in the open field test, percent time in the open arm of elevated plus maze, and sucrose preference to the same level as controls. Morrison et al. [74] compared isolated and social housing in adulthood after adolescent CVS. Contrary to previous findings in animal models of ELS, they found that CVS alone did not significantly alter any miRNAs across the transcriptome as compared with controls. However, CVS animals housed socially exhibited downregulation of 23 miRNAs [74]. These findings are consistent with studies that house animals in groups after ELS. However, it is still not clear why social isolation concurrent with CVS did not affect behavioral outcomes or miRNA expression. Finally, Liu et al. [76] applied an inescapable shock to chronically stressed adolescent rats and found decreased miR-135a expression in PFC and increased miR-16 in the hippocampus. To date, changes in miRNA expression in preclinical studies of ELS are not completely consistent. It seems clear that these changes are not only brain region specific, but they are also dependent on the type and timing of stress paradigm used.

### Clinical studies

The human literature on ELS as it relates to miRNAs is still quite limited and mostly overlaps with various psychiatric disorders. In an effort to explore the contribution of ELS to schizophrenia onset, Cattane et al. [77] collected blood samples from 32 control participants (11 with and 22 without early trauma history) and used microarray to assess miRNA expression levels in whole blood. Participants with any psychiatric diagnosis were excluded. Altogether, 80 miRNAs were found to be significantly differentially expressed in the early trauma group compared with participants without trauma [77]. Specifically, miR-29b-3p, miR-29c-3p, and miR-16-5p were significantly upregulated, while miR-200b-5p and miR-125b-1-3p were significantly downregulated. However, when they studied hippocampal miRNA expression in prenatally stressed rodents, they found decreases only in miR-125-1-3p, which was also present in cortisol-treated hippocampal progenitor cells [77]. This shows that miR-125-1-3p is specifically responsive to ELS and the effects are lasting and consistent across species. It also implicates hippocampal miR-125-1-3p in the corticosteroid signaling relevant to stress because of its response to cortisol treatment. In fact, miR-125-1-3p has been shown to target aldosterone synthase (*CYP11B2*) [78], the final enzyme in the conversion of cholesterol into the mineralocorticoid, aldosterone [79].

Since miRNAs themselves can be regulated through epigenetic modifications, two studies have explored methylation patterns on miRNA promoter regions in relation to ELS. In an all-male sample from low socio-economic status and high child abuse backgrounds, Suderman et al. [80] found patterns of altered methylation in promoter regions of 31 miRNAs. After confirmation using methylated DNA immunoprecipitation, they found that miR-514, let-7d, miR-520c, miR-215, miR-519a, and miR-519e were hypermethylated, whereas miR-203 was hypomethylated. Another study explored methylation patterns on the promoter region of miR124-3 in blood leukocytes from patients with borderline personality disorder (BPD) [81]. The results showed that a methylated region near the gene coding miR-124-3p was associated with severity of childhood trauma as well as BPD symptom severity. It should be noted that depressed patients without ELS were used as a comparison control because there was not an accessible BPD cohort without ELS exposure. These two studies are clearly limited in scope by their sample characteristics, nevertheless, they provide insight into the complex epigenetic changes induced by ELS exposure.

From the above described studies, it is clear that miRNA expression varies widely between different ELS paradigms and again across different species, with little overlap between studies. Still, seven miRNAs were identified by more than one study as relevant to ELS experience: miR-16, miR-18a, miR-9, miR-29, miR-200, miR-125, and miR-124. MiR-16 is upregulated in the hippocampus after both maternal separation [36] and inescapable shock [76] in rodents as well as in blood from healthy individuals with an ELS history [77]. In rodents, miR-16 has previously been implicated in serotonin transmission systems [82] as well as resilience to altered behavior after chronic adult stress [83]. Conversely, in a recent meta-analysis of human literature, Yuan et al. [84] found no significant differences in miR-16 expression between depressed patients and controls. Without comparing patients based on ELS exposure, it is unclear whether miR-16 contributes to depression after ELS. In addition, because miR-16 has been implicated in ELS, adult stress, and AD effects, it is not yet clear if miR-16 is a general marker for stress or if it is more specific to ELS.

MiR-124 has been widely studied in relation to MDD [40, 85] as well as GC function [86–88]. As previously described, stereotaxic injection of a miR-124 lentivirus into the hippocampus downregulates its target, *BDNF*, only in maternally separated animals [54]. Also, after adolescent CVS, miR-124 shows lasting increases in expression beginning in early adulthood [73]. Dexamethasone treatment yielded similar changes and RU486, a GR antagonist, negated changes caused by dexamethasone as well as CVS. MiR-124 expression after chronic unpredictable stress in later adolescence also correlates positively with severity of depression-like behaviors and inversely with GR expression in the amygdala [73]. A 180-min maternal separation also increased miR-124 expression as well as REST4, a key regulator of *BDNF* [52]. Lastly, in patients with BPD and a history of ELS, Prados et al. [81] found hyper-methylation of the promoter region of miR-124 was correlated with ELS history and symptom severity as compared with a sample of depressed patients with no trauma history. Together, this evidence supports the premise that ELS sensitizes different brain regions to miR-124 and alters GC pathway signaling, thereby causing depression- or anxiety-related behavioral outcomes. Furthermore, miR-124 interaction with *BDNF* may be mediated by REST4 during adolescent development. Though, it is well understood that miRNAs are responsive to early life environment, it is not clear how these external events precipitate change in miRNAs. Methylation is one candidate mechanism whereby miRNAs may be environmentally altered leading to subsequent changes in gene expression. In cortisol-treated rats, the Dwivedi group [40] found decreased methylation in the promoter region of miR-124 on chromosome 3 as well as decreased expression of DNA methyltransferase 3a concurrent with increased miR-124 expression levels and decreased target gene expression. Bearing in mind the sample limitations in Prados et al.—i.e., comparison of a no ELS MDD group to an ELS group with BPD—[81], further studies are necessary to explore miRNA methylation specific to ELS independent of psychiatric diagnosis. Thus far, miR-124 has been implicated in ELS [54, 73], acute stress [87], and MDD [40] as well as BPD [81], among others. Similar to miR-16, it is not yet clear how ELS and miR-124 uniquely contribute such different psychiatric disorders.

Currently, clinical findings in ELS are limited to studies of peripherally circulating miRNAs. Until the last 5 years, there have been almost no direct comparisons of miRNA profiles between the central and peripheral nervous system. In one study of patients with Alzheimer’s disease, it was estimated that 73% of 312 tested miRNAs were detectable in both cerebrospinal fluid and blood, but only 36 of these miRNAs were equally expressed [89]. In rodents that exhibited behavioral resilience to chronic mild stress, out of ten preselected miRNAs, miR-34a-5p was the only one found to be significantly upregulated in both serum and ventral tegmental area (VTA), but not prefrontal cortex [90]. Though the use of circulating blood miRNAs may be useful for biomarker detection, future studies will need to apply a systems neuroscience approach to identify viable therapeutic targets for psychiatric disorders.

## miRNAs in ELS-induced depression and schizophrenia

Although, miRNA changes relevant to depression are well documented in the literature [91, 92], no studies in MDD patients have explored the contribution of ELS experience to miRNA expression alterations. A recent meta-analysis showed that ELS interacts with an allele for FK506 binding protein 51 (FKBP5) to confer risk for depression or post-traumatic stress disorder [93]. Moreover, a SNP in the FKBP5 gene has been associated with susceptibility to develop depression after childhood physical abuse [94]. FKBP5 is a co-chaperone of GR, binding to GR in the absence of GC [95, 96] and, in increased concentrations, has been shown to compete for GR binding with corticosteroids [97, 98]. Normally, FKBP5 acts within cell nuclei to desensitize GRs after a stress response, thereby opposing the HPA response [95]. MiR-124 may play a role in altered FKBP5 binding and function via its interaction with GR. MiR-124 has been shown to target GR [40, 86] and relate to depression-like behaviors in animals models [73, 85]. Xu et al. [73, 75] not only found behavioral changes after adolescent CVS in rats, but also found increased miR-124 expression, decreased GR expression, and increased FKBP5 in the PFC, hippocampus and basolateral amygdala. Altered methylation patterns at the promoter region of miR-124 have also been found in patients with BPD and a history of early life adversity [81]. It is possible that ELS confers susceptibility to depression through these epigenetic changes involving miR-124.

Most often, animal models of ELS, including maternal separation and CVS, are intended as a depression model. Studies using animal models of ELS report increased behavioral phenotypes related to depression and anxiety, such as immobility in the forced swim test [52, 53] or anhedonia as measured by the sucrose preference test [52, 53, 73]. These behaviors are also reported to correlate with miRNA expression. O’Connor et al. [37] found that ketamine, electroconvulsive shock therapy (ECT), and chronic fluoxetine administration modified several miRNAs after early maternal separation, including miR-598-5p and miR-451; miR-9 was also downregulated, but only in ketamine and ECT treated animals. Clearly miRNAs are responsive to AD treatment after ELS, but it is still not certain what mechanisms are responsible for this effect. No studies have directly investigated the effect of ADs on miRNA promoter region methylation after ELS, but there is a strong relationship between AD response and global methylation patterns [99]. Specific miRNA expression alterations have been associated with resistance to ADs treatment, but little more is known about the mechanism of this effect [100, 101]. Further research is needed to determine if miRNA promoter methylation is a leading mechanism for AD efficacy, particularly in ELS.

Other psychiatric disorders, including schizophrenia, have been associated with ELS history [4] but little preclinical work involving miRNAs has been done in these disorders. A primary challenge for the field is to develop ELS-based animal models that approximate disorders other than depression or anxiety. Overall, less variety of miRNAs have been implicated in schizophrenia as compared with MDD [38] or even ELS. MiR-137, miR-181b, and miR-219-5p have consistently been identified in patients as peripheral signatures of schizophrenia regardless of ELS history [38]. In one clinical study of patients with schizophrenia and self-reported ELS history, miR-125-1-3p was significantly downregulated compared with patients without ELS [77]. Future studies in patients should utilize self-report measures like the Childhood Trauma Questionnaire [102] to conduct analyses both on diagnostic status and ELS history; similar methods have been applied to examine differences in brain function via fMRI [103].

## miRNAs in ELS and suicidal behavior

Suicide-related deaths are the most significant consequence of such prevalent MDD. Worldwide, an estimated 1 million people commit suicide annually [104]. Not only has suicide been attributed to depressed mood [105] or impulsivity [106], but early-life adversity has also been found to contribute [15] to increased suicide risk. Though several studies have investigated miRNAs relevant to suicide, no studies to date have assessed the contribution of ELS to miRNA profiles in suicide.

Although suicide is most often associated with depression, impulsivity, or a lack of self-control has also been found to predict suicide attempts [107] though this relationship is under debate [108, 109]. Teens who experienced earlier life stress report higher rates of impulsive behavior [110], and suicide rates among teens are higher than the general population [111]. Using an in-silico approach, Pietrzykowski and Spijker [112] identified several putative candidate miRNAs in the amygdala important for impulsive behaviors in mice, such as miR–190b, −28a, −340, −219a, and −49. A SNP in the binding regions of miR-641 on the *SNAP* gene has also been found associated with trait impulsivity [113]. In healthy individuals with an early adversity history, miR-641 was found to be significantly upregulated [77]. An interaction between this SNP and miR-641 may be involved in the relationship between ELS and later impulsivity. Several studies have shown changes in miRNA expression in depressed-suicide brain [39, 114, 115]. Specifically, miRNAs 497 [114], 146b-5p [115], and 330-3p [39] have all been reported in both depressed-suicide victims and healthy individuals with ELS history [77]. Further studies will be necessary to concretely link miRNA changes to both ELS experience and suicide or suicidal ideation.

## Conclusion and future directions

Epigenetic modifiers, especially miRNAs, have received increasing attention for their role in stress susceptibility after early stress. In the current review, we have presented evidence of altered miRNA expression after ELS experience. Table 1 lists all peer reviewed studies of miRNA expression after ELS in both animal models and human participants. Although, there was not much overlap in brain region of interest across the animal studies of ELS, some similar miRNAs were repeatedly found to be significantly altered. These miRNAs were also detected in blood of human participants with ELS history. Namely, miRNAs in the 124, 125, 29, 16, and 200 families were altered both in rodent brain as well as human blood. Rodent studies also reported a correlation between these miRNA expression levels and depressive or anxiety-like phenotypes. Collectively, the evidence suggests that ELS induces changes in miRNA function via a complex interaction of genes relevant to HPA axis as well as other neuroendocrine signaling systems.

**Table 1:** Preclinical and clinical studies showing involvement of miRNAs in ELS and associated depression and suicidality

Study	Species/model or human sample	Brain areas or source	Participating miRNAs	Reference
1	**Rat**, 180-min maternal separation combined with chronic restraint	Medial PFC	miR- 132, miR-124, miR-9, miR-29a	Uchida et al. [52]
2	**Rat**, 360-min maternal separation	Hippocampus	miR-16	Bai et al. [36]
3	**Rat**, 180-min maternal separation, fluoxetine, ECT, and ketamine treatment	Hippocampus	Fluoxetine: 2 miRNAs	O’Connor et al. [37, 33]
			ECT: 10 miRNAs	
			Ketamine: 14 miRNAs	
			All: miR-598-5p and miR-451	
4	**Rat**, 360-min maternal separation and adult chronic variable stress	Nucleus accumbens	miR-504	Zhang et al. [53]
5	**Rat**, 360-min maternal separation and adult chronic variable stress	Nucleus accumbens and striatum	miR-9, miR-326	Zhang et al. [59]
6	**Rat**, 90-min maternal separation and miR-124 lentiviral injection	Dentate gyrus	miR-124	Bahi [54]
7	**Mouse**, chronic variable stress	PFC	23 downregulated miRNAs including miR-200c	Morrison et al. [74]
8	**Rat**, inescapable shock	PFC and hippocampus	PFC: miR-125	Liu et al. [76]
			Hipp: miR-16	
9	**Rat**, chronic variable stress or dexamethasone treatment	Basolateral amygdala	miR-124a, miR-18a	Xu et al. [73]
10	**Rat**, 15-min maternal separation (augmented maternal care)	Hypothalamus	miR-488, miR-144, miR-542-5p, miR-421, miR-376b-5p	Vogel Ciernia et al. [69]
11	**Rat**, chronic variable stress or dexamethasone treatment	PFC and hippocampus	miR-124a, miR18a	Xu et al. [75]
12	**Rat**, prenatal restraint stress	Hippocampus	miR-125-1-3p	Cattane et al. [77]
13	**Human**, 40 men with high reported childhood abuse	Whole blood	Methylation at promoter region of miR-514, miR-520c, miR-215, miR-519a, miR-519e, mir-203, and let7d	Suderman et al. [80]
14	**Human**, patients with borderline personality disorder	Blood leukocytes	Methylation near promoter for miR-124-3	Prados et al. [81]
12	**Human**, 52 healthy adults characterized by childhood trauma score	Whole blood	80 miRNAs including miR-125-1-3p, miR-29b-3p, miR-29c-3p, miR-16-5p, miR-200b-5p, miR-641, miR-146b-5p, miR-497, and miR-330-3p	Cattane et al. [77]

Almost all brain miRNAs are coexpressed at varying levels across different brain regions [116] presumably according to a region’s functional needs. There is also evidence for cell-type (neuron vs glia) specific miRNAs in the central nervous system [117], which play a role in functions like neuronal differentiation and synaptic plasticity. Because ELS can have a profound effect on brain development (as compared with stress experienced in adulthood) and miRNAs have been strongly implicated in the process of neuronal development, ELS has the potential to significantly modify the anatomical distribution of miRNAs over time. For example, in ELS, neuron-specific miR-124 [117] was significantly altered in medial PFC, amygdala, and hippocampus [52, 54, 73, 75], whereas microglia-specific miR-200c [117] was altered only in PFC [74]. In the extant literature, animal models of ELS vary widely in terms of stressor type, duration, and developmental timing. Furthermore, few studies have explored the same brain region of interest, with the most studied brain region being the hippocampus. With such limited overlap across the literature, the connection between ELS experience and differences in miRNA expression—and, to a greater extent, function—is still not clear, although some of these studies have suggested that these miRNAs are involved in synaptic plasticity [52], dopamine receptor attenuation [59], and HPA-axis regulation [73]. Future replication is greatly needed in regions like the PFC, amygdala, and hypothalamus, which have also been shown to play a major role in the overall stress response as well as depression symptoms and suicide risk. In addition, it will be interesting to know how these brain areas coordinately regulate miRNA expression. MiR-124 may be of particular importance in ELS because of its role in neurodevelopment [118, 119]. Our lab has previously reported changes in miR-124 expression in PFC of corticosterone-administered rats [40]. Changes in miR-124 expression have also been reported after early postnatal stress [52], peri-adolescent stress [73, 75], and adult stress [87]. However, it has not yet been systematically tested whether miR-124 expression covaries with the postnatal developmental timing of stress exposure or other factors like chronicity, duration, or accumulation of stress. In particular, miR-124 has been implicated in synaptic plasticity along with miR-125b, −132, and −485 in dendritic morphology [120]. Although there is moderate overlap between miRNA affected by adult stress and those altered by ELS, unique changes will become clearer with further study. Rani et al. found that, in healthy adults, miRNA expression is positively correlated with age [121]. Going forward, it will be important to carefully consider the age of participants in miRNA expression studies, especially those examining the long-term effects of ELS. Considering the majority of the extant research has been limited to studying expression changes, it is also critical to examine the unique mechanisms by which miRNAs are altered after different types of stressors; here, we have presented miRNA promoter region methylation and circRNAs as candidate mechanisms, but the paucity of research on these mechanisms in ELS requires expansion and replication. Lastly, it would be valuable to explore differences in functions of miRNAs as a result of variation in stress. Several groups have used lentiviral injection to selectively augment and reduce individual miRNAs in living rodent brain [85, 122], including one ELS study [54]. Knockout mice have also been used extensively to study the importance of specific (and clusters of) miRNAs [123] and more recent technological advances propose non-invasive means of delivering brain-specific antagomirs to study miRNA knockdown [124]. In combination with genome-wide expression and in-vitro studies, these techniques can help us to elucidate the importance of miRNAs in stress susceptibility. Understanding miRNA characteristics specific to ELS as opposed to other life stresses will aid in designing more personalized treatment plans for those having depression or suicidal behavior.

Thus far, the human literature on ELS and miRNAs is limited. Future human patient and postmortem studies need to utilize available health history data or family report to determine if the patient experienced ELS. This could become increasingly possible as private health information is digitized and stored in national database. In addition, miRNAs isolated from blood present a challenge because these miRNAs represent a systemic expression profile, rather than brain-specific expression. Exosomes may prove to be the best candidate for isolating brain miRNAs from patient blood. Exosomes are small vesicles, packaged intracellularly in various tissues to move molecules, including miRNAs, extracellularly [125] and even across the blood–brain barrier [126]. These vesicle membranes contain proteins exclusive to their cellular origin. Fiandaca et al. [127] have used neural cell adhesion molecule L1 (NCAM-L1) antibody to immuno-precipitate neuron-specific exosomes from patient plasma. Although, a specific brain region cannot be identified for these peripheral circulating exosomes, this technology may help to identify brain biomarkers for disease susceptibility in living patients and may prove useful in prevention of disease onset after ELS. Ultimately, though, these methods still require validation in psychiatric patient populations. Initially, large scale and genome-wide studies of brain-derived exosomal noncoding RNAs will be particularly important for characterizing patients and their treatment outcomes. Because of their ability to contain and transport miRNAs and cross the blood–brain barrier, exosomes have even been proposed as a treatment vehicle [128]. In addition, it has been widely shown that current AD therapies can alter miRNA expression [129]. In the near future, providers may be able to use predictive algorithms to select AD treatments such that they target miRNAs either based on a diagnosed psychiatric disorder or even the individual’s circulating miRNA landscape as assessed by genetic testing. Although current research on ELS and miRNAs is limited, the exponential growth in both scientific technology and the storage of all types of health information makes it a promising avenue for neuropsychiatry study.
